# An automated tool for a uniform decentralized quality control and data analysis in multicenter studies with health care registry data

**DOI:** 10.23889/ijpds.v1i1.323

**Published:** 2017-04-19

**Authors:** Florian Endel, Christa Strassmayr, Heinz Katschnig

**Affiliations:** 1 IMEHPS.research

## Objectives

The EU FP7 funded project CEPHOS-LINK investigates hospital re-admissions of patients with a psychiatric disorder in 6 European countries by using linked health care registry data. In addition to the problems of different healthcare, payment and data collection systems, coordinating quality control, data analysis, and statistical modeling of sensitive data with six partners is challenging. For this purpose we have designed a secure online data analysis tool to diminish the time necessary to get results and incremental adaptions of reports as well as decreasing the chance and effects of misunderstandings between national and linguistic boundaries.

## Approach

A comprehensive study protocol clearly defining variables to be obtained and methods to be applied has been put together. The protocol is based on a thorough investigation of the different healthcare systems and related registries. It became clear that nonetheless misconceptions occur and the incremental improvements consumed vast amounts of available resources. Therefore a system which automatically creates the required reports including all tables, graphics and statistical models including data preparation based on a defined data structure has been developed. The report system is based on the statistical environment R and the document markup language LaTeX, tightly integrated with R's package "knitr".As this highly flexible solution is not straight forward to apply and implies various technical dependencies, a secure online platform hiding all technical details from the users has been developed. Utilizing state of the art software containers based on Linux and docker, a customized VPN solution, authentication and SSL encryption were put together. The web application itself is developed with R's "shiny" package and allows users to simply upload a dataset in the predefined format, interactively explore the contents, apply filters and generate the customizable, standardized report. Additionally, an offline version of the application is available for all major (desktop) operating systems.

## Results

The new platform advances data analysis and reporting in a situation where several partners are involved in analyzing local datasets, as is the case of the CEPHOS-LINK project. Integrating new features, graphics and research topics can be managed centrally while users can update their results and reports in nearly no time.

## Conclusions

The additional effort spent on developing a customized platform for quality control, data analysis and reporting has been worth the effort. Benefits include quick detection of implausible results, unifying the layout and graphics often depending on the software utilized and an established common data structure.

